# A Theoretical and Practical Treatise on Human Parturition

**Published:** 1849-03

**Authors:** 


### REVIEWS.

Art. IX.—*A Theoretical and Practical Treatise on Human Parturition.* By H. Miller, M.D., Professor of Obstetrics and Diseases of Women and Children, in the Medical Department of the University of Louisville. Louisville: John V. Cowling & Geo. C. Davies. Cincinnati: H. W. Derby & Co. New York: A. S. Barnes & Co. 1849. 8vo. pp. 463.

This work is no compilation. The author has aimed at something more than giving what others have taught and practiced in the art of obstetrics. His large experience has furnished him with the materials for forming his own independent opinions on all the unsettled questions relating to the art. These he has expressed, not dogmatically, but like one who had matured them carefully, and was thoroughly persuaded of their truth. Where the authors differ in reference to doctrine or practice, he has always his own decided convictions, for which it will be found that he has brought foward an array of arguments not easily resisted. Where, in his practice, he has not found things as others describe them, he is candid to acknowledge it. Where he has met with anything in writers which his experience does not approve, he has condemned it. His work is the result of long practice, and much reading and study. It is a judicious work. It may be criticised; on several points it is opposed to the highest authorities in the profession; but we are very greatly mistaken in our estimate of its character if any one, after a careful perusal, can lay it down without impressions highly favorable to the sound, discriminating judgment of the author. In his preface, he says: "To give a full, correct, and lucid description of the mechanism of labor, is a leading object which the author had in view, in writing this book. In the prosecution of it, he has not hesitated to examine rigorously and criticise freely the statements and opinions of others." His work he submits to the same scrutiny.

Prof. Miller believes, that, although many valuable works on midwifery have appeared in this country, one was still wanting on the subject—"a native authority to educe order out of confusion, and set up a national standard, under which all our practitioners may arrange themselves, and be cemented by a common bond of union." In regard to the fetal presentations and positions, our technology, as he remarks, has been thrown into confusion by the multitude of writers, and obstetricians find it impossible to communicate with each other in intelligible terms. His aim in the work which he now submits to his countrymen, is to carry them back to a standard upon which all may agree—"to review the whole subject of presentations and positions, with the light which his own experience and that of others has shed upon it, since the time of Baudelocque, and establish for them a classification and nomenclature which, it seems to him, all may adopt." He recommends the classification of M. Dugès; the nomenclature is his own. In the conclusion of his preface he avows that the principles upon which he has practiced midwifery are those of Hamilton and Burns, who regarded labor as "an exhausting struggle," and the office of the obstetrician as something more than an idle contemplation of the process. With this avowal, his readers will not expect to find him favorable to the expectant practice so fashionable since Denman's day. At the same time he is far from being obnoxious to the charge of a "meddlesome midwifery." He has, as appears to us, happily avoided the two extremes, indicating under what circumstances nature may be left to herself, and when, in her weakness, she demands the judicious ministry of the accoucheur.

Prof. Miller devotes the first four chapters of his treatise to the "rudiments of parturition," under which he embraces the *obstetric properties of the pelvis*, the *obstetric aptitudes of the fetus*, *the appurtenances of the fetus*, and the consideration of the *uterus as an organ of expulsion.* It is expected of the student that he shall make himself familiar with these "rudiments," and yet he will find no little difficulty in determining what they are amid the conflicting statements of authors on the subject. In regard to the obstetric properties of the pelvis, the exposition of M. Dugès, though the briefest of any writer, in Dr. M.'s opinion, is the best, and accordingly he adopts it.

In the second chapter, Dr. Miller examines at some length the question why the head of the fetus is so generally the presenting part. He shows the fallacy of the common belief, that it is owing to the higher specific gravity of the head. He refers to the experiments of M. Dubois, in which it was shown that when a fetus was plunged into water the head did not descend faster than the body. He also quotes the observation of this writer, that presentations of the head are proportionably less frequent in the earlier months of gestation, when the relative size of the cephalic extremity is greater. Finally, he cites the well-known fact, that in quadrupeds the head of the fetus presents with as much constancy as in the human species, when, on the physical hypothesis, it ought to be the furthest removed from the os uteri. The conclusion is, that the position of the fetus in utero depends, not upon physical, but upon vital laws; and there has been no better explanation proposed than that of

Dubois, that it is determined by the instinctive movements of the child made in obedience to an internal sensation—the principle, so strong in our nature, thus early exhibited, of seeking to improve our situation.

Of the appurtenances of the fetus the placenta is the one which has the most important office to perform. It is the organ of respiration and of nutrition. Through it the young being, during intra-uterine life, derives all its nourishment. Physiologists have perplexed themselves needlessly, as we think, concerning the change wrought by it in the blood. They have expected to find a change of color similar to that produced in the lungs by respiration, and have expressed surprise when they could detect scarcely a shade of difference in the color of the fluid circulating in the vein and the arteries of the cord. When we consider that the arterial hue is imparted to the blood by contact with the oxygen of the atmosphere, it is palpable that we ought not to look for it in the fluid leaving the placenta. For all the purposes of embryotic life, the blood of the placenta is sufficiently oxygenized. It is only after the fetus becomes independent and rises in the scale of being, that aëration of its blood becomes necessary.

The anatomy of the uterus is the subject of the fourth chapter, and it is shown that this differs materially in the different parts of the organ. Dr. M. adopts the doctrine that the body and neck are destined to perform different and antagonistic parts in the process of parturition. Before this commences they have different offices to perform. The cervix has no agency in the menstrual function, which belongs exclusively to the body. The body is for the reception, growth and accommodation of the ovum; the office of the cervix is to retain the ovum until the proper time arrives for its expulsion. The neck contributes nothing to the cavity of the impregnated uterus, the dilated body furnishing all the requisite space. The neck, therefore, "is the *sphincter* of the uterus."

"But the uterus, notwithstanding its natural division into body and neck, and its complete equipment of muscular fibers, would fail to fulfil its office as an organ of expulsion, were it not suitably endowed with *nerves*, to put it in relation with other organs, and enable it to receive and respond to impressions made upon it. It is accordingly supplied with nerves in great abundance, and from the two great centers of the nervous system, namely, from the great sympathetic and the cerebro-spinal axis."

The researches of Dr. Lee, to whom we are indebted for much of our knowledge respecting this interesting branch of anatomy, have fully established the fact, that the nerves of the uterus are enlarged by pregnancy.

We come now to the consideration of the efficient cause of *Labor*. What is it? We give our author's view of it:

"Labor, then, according to our view of it, is a contest between the body and the neck of the uterus,—the former aming to expel the fetus, and the latter to retain it. This is no novel doctrine; it was distinctly taught by the celebrated Levret, who maintained that, as the neck of the uterus is the antagonist of the body, during pregnancy, and serves to hinder the product of conception from being expelled, so the body is the antagonist of the neck, during labor, else the fetus could never escape; and if the neck prove too strong for the body, one of them must necessarily be ruptured."

The uterus becomes hypertrophied during pregnancy; its contents are expelled by its tonic contraction, together with the accessory action of the diaphragm and the abdominal muscles.

In the next chapter, the determinative cause of labor is considered. This, in the view of Prof. Miller, is "*irritation of the cervix, and especially of the os uteri, arising*

*from the contact of the ovum with it,*" a theory which he adopts from Dr. John Power. Like the bladder and the rectum, the uterus is stimulated to contraction by the pressure of its contents upon its sphincter. The arguments in favor of this doctrine are as follows:

"*First; The peculiar manner in which the uterine neck is unfolded during pregnancy.* It has been shown, in a previous chapter, that the neck of the uterus does not participate in the changes going on in the body, having for their object amplification of the cavity, but remains quiescent for a considerable time, and then undergoes changes peculiar to itself; that its unfolding, so as to admit the ovum into contact with it, is deferred to a very late period of pregnancy, until, in fact, a short time before labor sets in.

"*Secondly; The rectum and bladder being excited to expel their contents by irritation of their orifices, affords strong ground of presumption that the uterus is excited to action on the same principle.*"

But, continues Prof. M.,

"We need not dwell longer on presumptive evidence, when demonstrative proof of the truth of our doctrine is within reach. Here it is: *The uterus can be excited to expulsive contractions, especially in the latter months of pregnancy, by artificial irritation of its orifice.* This artificial irritation is established by the introduction of a finger within the orifice, and pressing upon its circle to stretch or dilate it. The knowledge of this fact led M. Puzos, a century ago, to propose his memorable innovation upon the previously-established practice in cases of flooding, occurring in the latter months of pregnancy."

We shall not follow the author through the lucid and philosophical account which he gives of the phenomena of labor, in his seventh chapter, but proceed to notice his treatment of its first stage laid down in the chapter succeeding.

There have been two schools, as is known, who have long divided the profession in regard to the management of the first stage of labor—the English and the Scotch. At the head of one stands Denman; Hamilton and Burns are the most noted names in the other. Denman held, that whether a short or a long time be required for the dilatation of the os uteri, the duty of the practitioner is not to interfere in the process. Hamilton held, that the termination of the first stage of labor ought to be secured within twelve or fourteen hours from its commencement; and Burns was disposed to abridge the time to ten, or, at most, twelve hours. Dr. Miller belongs to the latter school, and the reasons which he assigns for interposing in cases where the os uteri is slow to dilate, are such, we believe, as will carry conviction to most of his readers.

Among the causes of delay in the first stage of labor, are *obliquity of the uterus* and *inefficient action* of that viscus. The first may be generally remedied "by regulating the posture of the patient, enjoining her to lie on the side opposite that toward which the fundus inclines."

"Should strict attention to posture, continued for a reasonable time, fail to correct the obliquity, and the labor in the meanwhile make but tardy progress, it is proper *to hook the os uteri by inserting the extremity of the finger within its orifice*, and draw it toward the center of the pelvis, in the intervals of the pains. When a pain comes on, its tendency to relapse to its former position is to be resisted, with as much force as can be safely employed. If this tendency is too powerful to be resisted, the finger must yield to it; but, as soon as the pain ceases, bring back the os uteri to the center, and again endeavor to maintain it there during the next pain. By cautiously and gently, but perseveringly, acting thus, the os uteri will, after a succession of centripetal and centrifugal movements, be restored to its proper place, and the parturient forces having been brought to bear upon it, its dilatation will be effected as speedily as in ordinary cases."

For the second obstacle, if there be no morbid state of the system to account for the inefficient action, as fever, or confined bowels, Prof. Miller's remedy is *irritation of the uterine orifice by means of the finger.* In recommending this procedure, he is aware that he "stems the strongest current of authority that runs through obstetrics," but, sustained by the high sanction of Burns and Power, and by his own frequent experience, he insists, not only upon its perfect safety, but its prompt efficacy in bringing on uterine contractions. He gives the necessary directions for it in the following passage:

"In the first place the manner of doing this must be explained, in order to guard against any misapprehension or abuse of it. It is rarely necessary to employ more than the index finger, the extremity of which is to be introduced within the orifice, in the absence of pain, with its pulp or feeling surface turned toward the anterior lip of the os uteri. The patient is placed on her back, which is certainly the most convenient position for the manipulation. When a pain comes on, or after the lapse of the usual interval, whether there be pain or not, and for the purpose of exciting one, pressure is to be made with the finger, moved slowly around so as to bear successively on every part of the anterior semicircumference of the orifice. Having described a half circle in one direction, for example, toward the right side of the patient, the finger is to be moved in the same manner in the opposite direction, and these movements are to be continued during the pain, or, if there be no pain, for a minute or so. It is then to be withdrawn from the orifice, but retained in the vagina, or if kept within the orifice, it must rest from its work for a few minutes. When pain recurs, or should recur, the finger is to be used in the same manner, and so on, until the uterine contractions become stronger, and act with more efficiency upon the orifice, when it is to be withdrawn from the vagina. The invigorated contractions may finish the dilatation of the orifice without any further assistance. Should they flag, however, or progress slowly, the finger may be reintroduced, from time to time, to freshen them. If the membranes are ruptured, and the os uteri contracts much during a pain, there may not be room for the finger between it and the presenting part of the child: in this case the finger must be used, as directed, in the interval of pain, and when contraction comes on, it must be withdrawn and made to press on the verge of the orifice. To produce the requisite orificial irritation, the finger must press, with different degrees of force, in different cases; but, in all cases, the pressure should be gentle at first and gradually increased, and it is never allowable to use such force as would be required, literally, to stretch the os uteri. The mere contact of the finger is not sufficient to excite the os uteri; it is, therefore, necessary to make pressure with it, but it must be remembered that this pressure is intended to *stimulate*, not to *force open*, and that it acts upon a *vital*, not upon a *mechanical*, principle.

"Speaking from abundant experience, I can truly say that it is equally surprising and gratifying to observe the prompt effects of this manipulation, in many cases of the kind under consideration. Not unfrequently, a few movements of the finger are sufficient to impart such energy and aim to the uterine contractions, that the waters begin to gather, as the phrase is, and cause the membranes to protrude."

The subject is continued in the next chapter. A third cause which delays labor in the first stage, is *impeded action of the uterus;* and a fourth cause, is *morbidly resisted action*, or, as it is usually termed, *rigidity of the os uteri.* In cases of this sort, as in those just noticed, Prof. M. holds that it is the duty of the practitioner to act. Those of the cast of Job's physicians, he thinks, "sadly abuse the confidence of their patients"; and their patients, no doubt, if they understood the matter, would think with him. Bleeding, opium in the form of an enema, tartar emetic, are to be tried, with tepid baths, and the topical application of extract of belladonna and stramonium. And, finally,

"Should all the means above recommended fail, or but partially succeed, in overcoming the rigidity of the os uteri, and the cervix descend in advance of the head of the child, it is necessary *to raise and support the os uteri.* As this is a measure of considerable importance, I shall endeavor to explain how it is to be practiced. The index finger is to be applied just underneath the anterior lip of the os uteri, and with its edge or palmar surface pressure is to be made, in the interval of the pains, so as to push up the os uteri as high as possible, or the extremities of two *or three of the fingers may be used* in the same way. When a pain comes on, the tendency to descent is to be resisted, unless this be so strong as to require more force than it would be prudent to employ: in that case, the finger or fingers must gradually relax its counter pressure and allow the descent to take place. But as soon as the pain goes off, the os uteri is to be pushed up again, and its descent is again to be resisted during the next pain. In this manner, acting mith gentleness and caution, but, at the same time, with firmness and perseverance, the os uteri must be supported until it is sufficiently dilated to allow the head to execute its rotatory movements, and emerge from under the symphisis pubis.

"The principle upon which this maneuver acts does not appear to have been well understood, even by those who have practiced it. The support given to the os uteri prevents it from prolapsing, to be *backed*, if the expression will be allowed, by the floor of the pelvis, and places it in a position that will permit a portion of the head to become insinuated within it during a pain. The finger is not used to stretch the os uteri, as many writers direct, but to hold it up that it may be dilated by the head, which can then be pushed, by the uterine contractions, lower than the level at which the os uteri is held. The head dilates the os uteri far better than the finger could, because it acts upon the whole extent of the cervix, whereas the finger could act only on the circle of the os uteri."

Describing the common phenomena of the second stage of labor, Dr. M. remarks at some length, towards the close of the chapter, upon the changes of the uterine circulation, and shows that if this stage be protracted by any obstacle requiring unusually powerful efforts of the uterus to overcome it, the death of the fetus is apt to occur. At every labor-pain of great intensity an interruption of the circulation must take place; the fetal respiration is thereby suspended, and if this continue beyond a certain period, the fetus must be born asphyxiated. We shall see how our author brings these principles to bear upon the use of ergot further on.

The treatment of this stage of labor forms the subject of the eleventh chapter. Uterine hemorrhage is the chief danger attending this stage, and a frequent cause of it is the too rapid progress of the labor—"the expulsion of the child immediately following the rupture of the membranes." The tonic contraction has not time to effect the proper change in the volume of the uterus, and if the placenta "should be detached, in whole or in part, by the muscular contraction that expelled the fetus," hemorrhage must ensue. The remedy is to rupture the membranes in time; viz: "*when, the dilatation of the os uteri being completed, the pains become expulsive, or even in the absence of expulsive pains and with a view to excite them.*" This practice is generally condemned by obstetrical writers; but, with our author, we believe that it is the true practice. The membranes have performed their office when they have dilated the os uteri, and then, if they do not give way themselves, they ought to be ruptured.

This stage may be protracted by "inefficient" or by "impotent action of the uterus." For the first, bloodletting is sometimes resorted to with advantage, and sometimes castor oil, or a stimulating enema; but the great remedy is *ergot*, the conditions favorable to the use of which are laid down in all the authors. The remedy, Dr. Miller thinks, is far from being a safe one. He remarks,

"From the exposition that has been made of the changes induced in the uterine circulation, by the parturient contractions, it is evident that if these contractions were not alternated with intervals of repose, the fetus would be inevitably destroyed, in every case of parturition, before its expulsion could possibly be effected. Such unrespited contractions of the uterus, as we have supposed, are, it is very well known, produced by ergot; when it is exhibited and takes effect fully, the uterus is urged to one long and unceasing effort until its contents are evacuated. A radical change is, therefore, induced in the mode of uterine contraction, which is tantamount to wresting the process of parturition from the hands of nature. No respite from her sufferings is allowed the mother,—no breathing time for the fetus. What wonder, then, if the former is more exhausted by the labor, and the latter ushered into the world completely asphyxiated,—its countenance swollen and livid, and its vital organs engorged, or oppressed with extravasations of blood!"

This violent and unintermitted contraction may, moreover, produce fatal compression of the child's brain, as our author adduces instances from other writers to show. He continues, on the same subject—

"But the destructive tendency of ergot, as already intimated, is not limited to the child. When prematurely or incautiously administered, it may cause rupture of the uterus, by goading it to exert a degree of force imcompatible with its integrity, but yet insufficient to overcome the obstacles that may oppose it. Especially is this true in cases of disproportion between the size of the child and that of the pelvis, and when there is unusual resistance of the os uteri and perineum."

In proof of this Dr. Miller cites the following cases. He says,

"That rupture of the uterus has been often caused by the exhibition of ergot, I cannot doubt, after a careful examination of cases that have been reported in the medical journals. One journal published in this country, in a single month, (April, 1841), not to refer to others, contains two cases, in which fatal rupture of the uterus was owing to the use of ergot, though the true cause of the accident does not appear to have been suspected by the reporters. It may be useful to give an abstract of these cases,—premising that the title of the journal, referred to, as well as the names of the parties concerned, are suppressed, lest offense should be given, and I be accused of a spirit of ill-natured personal criticism, which does not at all actuate me.

"The first occurred in the practice of ——, at the time one of the editors of the journal. The patient, who had given birth to a dead child two years previously, was taken in labor about four o'clock, P. M., Friday, 16th March; seven hours afterward the os uteri was well dilated, the head presenting in the first position; the protrusion of the scalp indicated its compression on account of the small size of the pelvis, and but slow progress was made for the next five hours; the head became jammed in the pelvis, and was stationary for the next eight hours, when twenty drops of the wine of ergot were administered 'in the hope that more efficient contractions of the uterus might perhaps mold the head of the child to the cavity in which it was impacted:' the ergot having no effect, in an hour and a half, an attempt was made to deliver with the forceps, which failed on account of the pelvis being so absolutely filled up with the head, that 'it was not possible to introduce the smallest-sized catheter into the bladder:' the patient was next bled and *got a scruple of powdered ergot;* this was at four o'clock, and at six, *the pains being strong*, forty drops of laudanum were given, shortly after which *the alternate contractions of the uterus entirely ceased*, and she was attacked with *pain in the epigastric region and vomiting;* an attempt to deliver with the forceps was again made, with no better success than at first, when it was determined to deliver by embryotomy, which was accomplished with great difficulty, and 'literally with main force.' It is not necessary to pursue the subsequent history of the case; suffice it to say that the patient died, and a post-mortem examination revealed the existence of rupture of the uterus, with extravasation of blood into the abdominal cavity, and intense peritoneal inflammation."

"The second case, entitled 'Utero-Vaginal Rupture,' is communicated by ——. The patient was in labor with her third child, and had complained of slight pains during the whole of the day before the doctor was called: the os uteri was found to be but partially dilated, although it was very easily dilatable; he ruptured the membranes, which failing to improve the labor, ten grains of ergot were given with the effect of increasing the pains, but these soon beginning to languish, the dose was repeated, shortly after which she had a *few very severe pains.* The action of the uterus then gradually declined, and the os uteri became less dilatable, for which it was judged proper to bleed her, but *she did not bear the abstraction of blood well*, the loss of eight ounces rendering her pulse 'weak and frequent'; four hours after the bleeding the os uteri was more dilated, but the pains were still weak and inefficient; an anodyne was given, and she was left for the night; at nine o'clock next morning it was reported that she had passed *a very restless night*, and had complained much of pains, but few of which the patient thought efficient: on examination it was found that the os uteri was fully dilated, the breech presenting, and advanced three-fourths of an inch to an inch lower than at last examination: ineffectual attempts were made with the hand to bring down the feet, and ergot was again administered, but did not produce the least effect. It was now determined to deliver with instruments; the feet were with difficulty brought down with the blunt hook; the body was soon expelled and the arms brought down, but when the head came into the pelvis the patient was so much exhausted, that it was deemed prudent to defer the extrication until the patient was revived by stimulants; the head was then delivered with the crotchet, but not without extreme difficulty, and there was not the least subsidence of the abdominal tumor until the delivery was nearly completed. It is needless to follow the further details of the case: the patient died the next day after delivery, and the autopsy disclosed metroperitonitis, effusion into the abdominal cavity, with coagula of blood, and transverse rupture of the left side of the neck of the womb, extending into the vagina."

Certainly, we think with Dr. Miller, there is no room to doubt, that "goading the uterus with ergot" was the cause of the fatal issue in these cases. It was, assuredly, wholly inadmissible under the circumstances; but, as Dr. M. remarks, it is not always an easy matter to decide with confidence that all the conditions exist which justify the exhibition of ergot, and it is therefore fortunate that the obstetrician has other resources.

"The manipulations, which I have recommended in the first stage, may also be usefully employed in the second, with marked effect. The anterior margin of the os uteri, although it be sufficiently dilated to allow the fetus to pass, may be commonly felt behind the pubes, and the finger is to be insinuated between it and the presenting part of the child, in the absence of pain, and press it upward, performing, at the same time, semicircular movements, as already explained. When a pain comes on, the finger is still to retain its place and continue its operations, unless the cervix contract with such force as to compel it to give place, in which case it is to be withdrawn, but be reintroduced when the pain subsides, and act as before."

When the head has descended so low that the os uteri cannot be felt, still by pressure with the finger on the posterior wall of the vagina the expulsive contractions of the uterus, etc., may be aroused. In recommending this procedure Dr. M. has before his mind the ridicule of that large party of accoucheurs who discredit the value of all such manipulations; but he is unmoved by it, convinced as he is, by much practice, of its utility.

*Impotent* and *inefficient* action of the uterus are not the same thing. In the former, the force is inadequately exerted because the ability to act is destroyed; in the latter, it is not exerted though it exists in full vigor. In cases of impotent action, it is desirable to ascertain whether the child be alive or not, and this is to be done by auscultation.

"On applying the ear to the abdomen of a woman in advanced pregnancy, if the fetus be alive, the action of the heart can be discovered by the sounds emitted from it. These sounds, compared by Kergaradec to the ticking of a watch, may be recognized as cardiac by their resemblance to those of the adult heart, with which they cannot be confounded, on account of their much greater frequency. The action of the fetal heart is much more easily discoverable in the second stage of labor, because the liquor amnii being discharged in whole or in part, it is brought nearer to the walls of the mother's abdomen. The examination being made in the interval of the pains, a practiced ear will not unfrequently hear the cardiac sounds, the instant it comes in contact with the abdomen.

"If it be discovered that its heart is in action, the evidence of the life of the child is, of course, complete. But if we fail to make this discovery, it has been questioned whether the evidence of its death is quite as satisfactory. The proof is, it is true, of a negative character, and it is certainly possible that the child may be alive; and yet, owing to its peculiar situation in the uterus, we may not succeed in detecting the action of its heart. I cannot but believe, however, that if a careful examination of every region of the abdomen, occupied by the gravid uterus, discloses no trace of the heart's action, (supposing, of course, that the examiner has the requisite experience and tact), the fetus has lost its vitality, or at least the probability of this is too great to justify us in adopting any method of delivery, based on a contrary supposition. The fetus is, therefore, dead to all *practical* intents and purposes. Under certain circumstances, the cessation of the cardiac sounds is conclusive proof of the child's death as occular examination could afford; I mean where, in the early part of labor, we can distinctly hear these sounds, but discover that they grow less and less audible, as labor progresses, until they become entirely extinct. My own practice is to seek an early opportunity to apply my ear to the abdomen, in every case of labor; and then, if the labor should be protracted, I have a sure index of the child's condition, which should always be taken into estimation, when we are deliberating as to what measures shall be adopted."

In the treatment of uterine impotency, the remedies so useful in inefficient action are of little avail. Blood-letting, ergot, and the manipulations with the finger cannot be relied upon in this condition of the uterus.—What then is to be done? In the end, our resource is generally instrumental delivery, the period of resorting to which must be left to the judgment of the practitioner.

We have said that in respect to *presentations*, Prof. Miller adopts the classification of M. Dugès, exhibited in the following synopsis, in which is also shown the frequency of the different presentations in 37,126 cases of labor:

| *Genera.* | | *Species.* | | | *Frequency.* |
|---|---|---|---|---|---|
| I. Vertex; | 35,375 | 1st. | Back | anterior and left; | 27,443 |
| | | 2d | " | anterior and right; | 7,512 |
| | | 3d | " | posterior and right; | 276 |
| | | 4th | " | posterior and left; | 144 |
| II. Pelvis; | 1,390 | 1st | " | left; | 854 |
| | | 2d | " | right; | 494 |
| | | 3d | " | anterior; | 14 |
| | | 4th | " | posterior; | 26 |
| III. Face; | 175 | 1st | " | left; | 99 |
| | | 2d | " | right; | 76 |
| IV. Right shoulder; | 103 | 1st | " | anterior; | 57 |
| | | 2d | " | posterior; | 46 |
| V. Left shoulder; | 83 | 1st | " | anterior; | 52 |
| | | 2d | " | posterior; | 31 |
| 5 | | 14 | | | 37,126 |

The nomenclature proposed by Prof. M. strikes us as preferable to that of M. Dugès. A nomenclature ought to be agreed upon by writers and teachers in America, and unless one can be devised less exceptionable than that which he here proposes, we hope it will be adopted.

Under the head of the phenomena of the second stage of labor, the subject of the fourteenth chapter of his work, Dr. M. presents a most lucid and satisfactory description of all the steps of the process. Having furnished what he considers a correct description of it, in all the vertex positions, he proceeds to discuss the etiology of the rotation of the head, which has given rise to a greater diversity of views than almost any other phenomenon in the process. He concludes the discussion with his own view of the matter, and remarks:

"I feel bound to declare my own unshaken faith in the reality of such a rotation as I have ascribed to the head, in the account already given of the mechanism of labor. This faith does not repose upon authority only, but, as I flatter myself, upon careful and repeated observation. Like Nægelé, I have in numerous instances watched the different evolutions of labor, with my finger, for hours, now on a fontanel, and anon on the commissures, and if I have not almost uniformly verified the *complete* rotation of the head, I have as yet learned nothing in midwifery. When, in a first position of the vertex, the posterior fontanel is, at a certain stage of labor, plainly felt behind the left obturator foramen, while the lambdoidal commissure is to the left of the symphysis pubis, and the sagittal crosses the vagina obliquely from above downward and from left to right; but in the further progress of labor, the lambdoidal is brought completely forward, so that its branches cross the rami of the pubes equidistantly from the symphysis, and the sagittal runs vertically, parallel with the genital fissure, while the posterior fontanel is to the left on the same line,—when, I say, all this is plainly and nearly invariably felt, the conviction must be riveted upon the mind of the observer, that *the head does verily completely rotate.* Such is my experience—or, if it prove unreal, my dream, for more than twenty years."

In vertex presentations the accoucheur has generally little to do; "nature must be allowed to do her work in her own good way." There are, however, not a few cases in which the passage of the head and shoulders may be facilitated by the proper assistance.

"Let us inquire, then, what may be safely done to favor the release of the head, when it is pressing on the perineum, but its exit is unduly delayed. The delay may arise from the resistance of the soft parts at the outlet, or from the inadequacy of the parturient powers to cause the head to execute the movement, by virtue of which it clears the vulva. The execution of this movement (extension), it has been shown, requires a degree of uterine force, of which they alone have any just conception, who have carefully studied the mechanism of labor. In either case, firm and properly directed pressure on the perineum will avail much in promoting the birth of the head, by aiding the movement in question. The pressure should be, of course, from the extremity of the sacrum toward the symphysis pubis, and so managed as not only to push the forehead of the child in that direction, during a pain, but as much as possible in the intervals of the pains, so as to retain whatever advantage is gained. The patient lying on the back, both hands, with the extremities of the fingers directed toward the sacrum, may be employed in raising the head, as it were, toward the pubes. By acting thus, I have often succeeded in having the head expelled by a few pains, notwithstanding it had made no advance for hours previously, and there appeared to be no prospect of its expulsion by the unaided offorts of nature. Of Baudelocque's maneuver, slipping a finger or two under the jaw, much less of Mauriceau's, laying hold of the head, for the purpose of making traction upon it, I have no experience."

Directions are also given for the extraction of the shoulders, for the management of the umbilical cord when it is found encircling the neck of the child, and for the far more serious impediment to delivery, the permanent contraction of the cervix uteri about the child's neck. For the latter,

"The treatment consists in the dilatation of the contracted portion of the uterus by the fingers, insinuated between it and the back of the child. This can be accomplished only by first elevating the head above the superior strait, for there is not room in the excavation to receive the hand between it and the pelvic walls. When, therefore, the diagnosis is established, in the manner already explained, we should proceed at once, without withdrawing the hand, to remedy the difficulty. The stricture being dilated, the hand should be suddenly withdrawn, upon the access of a pain, that the shoulders may take its place, and prevent a recurrence of the accident."

Capital blunders have been committed by experienced practitioners in such circumstances, and it therefore behooves the young accoucheur to be cautious in his diagnosis. He is exposed to the risk of mistaking the case for one of impotent action of the uterus, and of resorting, improperly, to the use of instruments. Smellie reports a case in which he fell into this error.

Chapter XVI is devoted to "instrumental delivery in vertex presentation," and the various operations pertaining thereto are described with clearness and precision. The subject is a grave one, and our author takes occasion to show that grave errors have been committed by eminent writers who have described, as well as by practitioners who have attempted to execute, the several maneuvers in instrumental delivery. If the directions given in this chapter are heeded, we shall cease to hear of that "barbarous midwifery" of which "fistulous communications between the bladder or rectum and vagina" are the legitimate fruits.

The next chapters treat of "nates presentation," in which, as in cases of vertex presentation, nature is generally to be left to accomplish the process unaided. "*Natural delivery*," Dr. M. says with emphasis, "in *all cases*, is *preferable to artificial*, but in none more than in nates presentation." It is not, in every case and under all circumstances, an easy matter to make out the diagnosis of this presentation. "The anus," it has been said, "is so characteristic, that it ought to be sufficient to prevent mistakes." To which our author replies,

"Aye, so it ought, if it were always found a closed and puckered orifice; but if the child be dead, and a curious examiner have been poking at it before we are called, it may be gaping and tumid, and feel like the mouth, while the buttocks, to the touch alone, are not unlike the cheeks. No wonder then if it should be mistaken for a face presentation, one instance of which is within the compass of my own knowledge,—in sooth, *magna pars fui.*"

In "face presentations" turning has been proposed by high authority. Dr. M., very justly as we think, condemns the practice, which, "with the single exception of the Cæsarian section, is the most artificial of all the modes of delivery." We are neither "to redress the head, nor turn, nor use the forceps, merely because the face presents;" no special treatment is demanded, but we should exercise "all reasonable confidence in the ability of nature to accomplish what she proposes."

We come next to a presentation of a very different character, that of the shoulder. But even in this apparently hopeless case, the expulsion of the fetus has sometimes been effected by the unassisted efforts of nature, an instance of which is related by Dr. Miller. He says,

"February 27, 1846, I was requested by Dr. Donne, now one of the professors in the Memphis Medical College, to accompany him to the house of Mrs. B., on Market street, who was in labor with her second child, under the disadvantages of a shoulder presentation. I found Dr. Lewis Rogers at the house, who had made an effort to turn, but was defeated by the strength of the uterine contractions. It was plain, from the patient's behavior, that the pains were still exerted with unusual vehemence as well as frequency, and I proceeded, as soon as possible, to make an examination; when it was discovered that the left shoulder presented in the second *(scapulo-sacral)* position, with the arm extended and the hand protruding through the vulva. Before the examination was completed, however, the perineum began to be distended, and I remarked to the medical gentlemen, that the child would probably be expelled by duplication, which did accordingly occur in a few moments afterward, in the manner already described. The child, which appeared to be fully developed and of average size, was born dead; its left arm being considerably swollen, and the left side of the neck, with the corresponding cheek, retaining marks of the contusion they had suffered. The labor was not unusually protracted, and the mother recovered without any unfavorable consequences. It should, perhaps, be observed, that the time when this case happened seemed to be propitious for *independent* childbear ing; as no fewer than four women, to whom I was called, were delivered by dame Nature, before I could reach their domicils, albeit I made as much haste as is consistent with obstetric dignity."

We need not follow the author through the directions he gives for the relief of such cases. They will be found precise and judicious.

In the next chapter we have the "phenomena and management of the third stage of labor," which com prises, as all understand, the separation and expulsion of the secundines. Believing that the placenta is detached by the tonic and not by the muscular contraction of the uterus, he is not anxious for a return of pains after delivery, but, when he finds the organ gathered into a ball under his hands, proceeds, ordinarily, to deliver the secundines in the absence of them. He says,

"For my own part, I am ready to avow that I seldom wait for pains, or inquire of the patient whether she feels them or not,—my only solicitude being to have the wound contracted, and the placenta naturally separated. These conditions existing, I proceed, without delay, to extract the placenta, like a good *miller* (pardon the pun), when its turn to be served comes, that is, after the matters, already specified as entitled to precedence have been dispatched."

The subjects of the last chapter are "asphyxia neanatorum, morbid retention of the placenta, and uterine hemorrhage before and after the removal of the secundines."

Asphyxia in the newborn child, Dr. Miller has shown, is often the consequence of the administration of ergot to the mother. "The bowstring," he says emphatically, "is not more murderous than ergotic contractions of the womb;" and we see no reason to controvert the truth of the remark. In the unyielding pressure of the womb upon the fetus, under the stimulus of this article, the placental circulation must cease, and with it all the functions dependent upon vitalized blood. While this derangement is kept up, the action of the heart is found to grow weaker and weaker, until it is no longer perceived. The asphyxia may be simple or apoplectic. By most writers, the latter condition is treated of under the name of apoplexy. In this, while there is insensibility, immobility, and absence of respiration, as in the simple variety, the heart and umbilical arteries may continue to pulsate, and there is also "greater lividity and swelling of the face, the eyes being prominent, and injected with blood, and the pupils dilated." As to the cause, Dr. Miller remarks,

"Apoplectic asphyxia, although it may result from the same causes as the simple, is, I apprehend, most usually induced by severe and long-continued compression of the head, from difficult and instrumental deliveries,—whereby the blood is forced from the surface of the head to the brain, by pressure of the jugular veins, in face presentations, or by compression of the inferior parts of the body, in nates presentations, particularly where the feet are foremost, in consequence of which the blood is determined to the brain, because it is excluded from the lower parts."

The treatment will vary in the two forms. For the first—

"Simple asphyxia is to be remedied by the employment of all the means calculated to put the respiratory apparatus in motion. Among these, one of the most powerful is, *sprinkling the surface, particularly the face and chest, with cold water.* For this purpose, the fingers should be repeatedly dipped in cold water, and shower the fluid, with considerable force, upon the parts indicated. The practitioner ought to be careful not to have the fingers too wet, so as to drench and chill the child, and after each application, or, at all events, now and then, the surface should be wiped dry and well rubbed. The skin is thus rendered more sensitive, and the probability is greater that the respiratory nerves will be excited through its medium."

With the same view, frictions are to be made on various parts of the body, the palms of the hands and soles of the feet to be excited by slapping them; and in addition camphorated spirits, or ammonia may be applied to the nose, and a little camphorated spirit put in the mouth. These details, though they may appear trivial, are not to be slighted by the young accoucheur. His success in resuscitating the child will often depend upon the address and perseverance with which he uses these appliances. Having directed them all in vain, the only remaining resource is artificial inflation of the lungs, to be accomplished by applying the mouth to the child's mouth and expiring the air pretty forcibly, "taking the precaution to close its nostrils with the fingers of one hand, while those of the other press moderately upon the trachea, to close the esophagus," and insure the passage of the air into the lungs. These various efforts are not to be hastily relinquished, for in some instances Dr. Miller has known them to succeed, "after the lapse of half an hour of disappointment."

The abstraction of blood is indicated in some cases of simple asphyxia.

"Whenever the child is decidedly plethoric and congested, although the congestion may not reach the height of apoplexy, it is useful and salutary to detract blood from the cord. Nay, ample experience justifies me in saying, that, when even simple asphyxia does not exist, if the child be plethoric and discolored, showing that it has suffered from the manner in which it has been ushered into the world, it will be benefited by losing a little blood, and secured, as I have reason to believe, from the convulsive affections, inflammations, and hemorrhages, to which it would be otherwise obnoxious."

So much for the first variety.

"In the apoplectic form of asphyxia, the great remedy is *bloodletting*, which must be promptly practiced by cutting the umbilical cord, when, if its circulation be active, blood immediately spouts from its arteries. We have been directed to receive the blood in a vessel or upon a diaper, that we may estimate the quantity subtracted. But I take no such precautions, and instead of looking nervously at the blood, look at the child's countenance, watching the chasing away of its purple hue by the rosy tints of health; and when its complexion is good, I arrest the bleeding. While the blood is flowing, the child usually begins to breathe, at first with a sort of convulsive struggle, but presently it breathes deeply, and announces, by its cries, that it has escaped the danger that menaced it at the portal of life. If the umbilical pulsation be faint or extinct, blood cannot be procured so freely from the cord; when the bleeding should be promoted by stroking it and immersing the child, to its naval, in warm water, made more stimulating by the addition of salt or mustard. If blood cannot be obtained in this way, a leech should be applied behind one or both ears. If bloodletting fail to resuscitate, the other means, already recommended in simple asphyxia, should be tried."

Morbid retention of the placenta may result from atony of the uterus, from a morbid adhesion, or from irregular contraction of the womb. In cases of the first there is no uterine excitement so powerful as the introduction of the hand into the flaccid cavity. Dr. M. declares in reference to the operation,

"My own 'fixed' rule of practice, in this case as well as in retention from the other causes mentioned, is, to have recourse to this manipulation, in an hour after the birth of the child, it being understood, of course, that all other means have been diligently but vainly tried within the hour. The passage of the hand, well lubricated with oil or lard, along a track so recently traversed by the child and yet patulous, from atony, is neither a painful nor difficult operation."

Our limits require us to close here our notice of Prof. Miller's volume; nor indeed is it needful that we should extend it further. Most of our readers are among the friends and pupils of the author, and will lose no time to possess themselves of a copy of his work. We venture to predict that it will prove equal to their expectations, and that it will give him a high rank among American medical authors. Its style is generally unexceptionable. An occasional sin against our great lexicographer, a word, now and then, not found in the dictionaries, and a single pun, however they may appear as blemishes upon a book for the most part elegantly written, are not to be set down as serious drawbacks upon its literary merits.

